# Paracolpium Leiomyoma Misdiagnosed as Endometriosis: Case Report and Surgical Technique of a Laparoscopic Approach

**DOI:** 10.1155/2022/7931391

**Published:** 2022-05-02

**Authors:** Nilton de Nadai Filho, Claudio Peixoto Crispi Junior, Marlon de Freitas Fonseca

**Affiliations:** ^1^Ministro Costa Cavalcanti Hospital, Foz do Iguaçu PR, Brazil; ^2^Crispi Institute of Minimally Invasive Surgery, Rio de Janeiro, RJ, Brazil; ^3^Fernandes Figueira National Institute for Women, Children and Youth Health, Rio de Janeiro, RJ, Brazil; ^4^Department of Women's Health-Fernandes Figueira National Institute for Women, Children and Youth Health-Oswaldo Cruz Foundation, Rio de Janeiro, RJ, Brazil

## Abstract

A 36-year-old Caucasian, nulliparous patient sought care at a private gynecology clinic after 6 months of attempting to conceive. During the initial consultation, the patient reported severe dysmenorrhea and deep dyspareunia. During the gynecological examination, a nodule in the left lateral vaginal fornix was palpable. The MRI showed a hypointense nodular lesion in the left paracolpium described as an endometriosis nodule. Laparoscopic resection of the nodule was indicated. The patient showed improvement in symptoms after surgical treatment. This case report describes the technique for laparoscopic approach to paracolpium tumors.

## 1. Introduction

Uterine leiomyomas are very common in women during menacme [1]. Vaginal leiomyomas, in contrast, are considered rare [2]. The most common location of vaginal leiomyomas is the anterior vaginal wall, but there are reports of leiomyomas in the lateral vaginal wall (paracolpos) and in the posterior vaginal wall (rectovaginal septum) [3]. Vaginal leiomyomas can be asymptomatic, but depending on their location and size, they can cause urinary symptoms (frequency, hesitancy, urinary retention, and dysuria), premenstrual discomfort, dyspareunia, or symptoms consistent with vaginal prolapse [3, 4]. In most cases of vaginal leiomyomas, there is no change in the vaginal mucosa. When a vaginal tumor is associated with ulceration, bleeding, or purulent discharge, the possibility of malignancy should be considered [1, 3].

Due to the low incidence of leiomyomas in the paracolpos, other diagnoses should be considered when a mass is observed in this anatomical region [2, 3]. Magnetic Resonance Imaging (MRI) and Transvaginal Ultrasonography (TVUS) are the principal imaging tools used in the workup of pelvic masses. The definitive diagnosis, of course, is anatomopathological.

For patients whose paracolpal tumors are asymptomatic, initial management can be expectant as the probability of malignancy is low [2]. In the present case, however, surgical resection of the paracolpal mass was indicated because the patient had severe dysmenorrhea and dyspareunia.

## 2. Case Description

This case report was approved by an institutional review board, the Research Ethics Committee (CAAE 44315920.9.0000.8527). Written patient consent was obtained and is on file at our institution. To improve transparency and reporting quality, the SCARE Guideline (consensus-based surgical case report) was used as a checklist (http://www.scareguideline.com).

A 36-year-old Caucasian, nulliparous patient sought care at a private gynecology clinic after 6 months of attempting to conceive. During the initial consultation, the patient reported severe dysmenorrhea and deep dyspareunia. On a self-reported 11-point numerical rating scale (NRS) in which 0 means no pain and 10 means terrific pain, the scores were 7 for dysmenorrhea and 10 for deep dyspareunia.

The patient reported no comorbidities and denied previous surgeries. During the gynecological examination, palpation of a nodule in the left lateral vaginal fornix reproduced the patient's dyspareunia. On specular examination, the nodule did not bulge the vaginal wall; no changes in the vaginal mucosa such as ulceration or bleeding were observed.

### 2.1. Preoperative Assessment of the Pelvis

**Figure 1:**
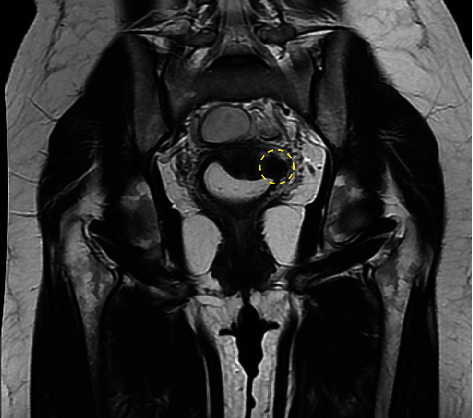
Coronal T2-weighted MRI. Tumor in the left paracolpium demarcated in yellow.

**Figure 2:**
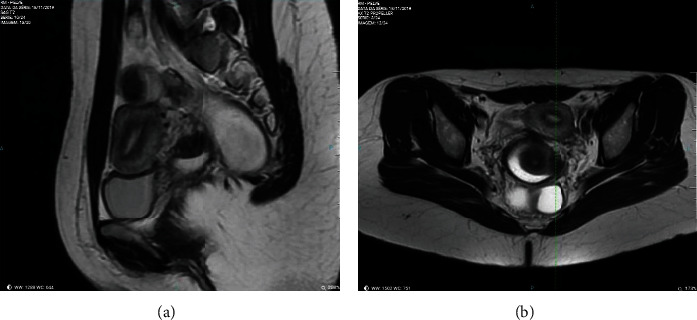
(a) Sagittal T2-weighted MRI. (b) Axial T2-weighted MRI.

The ovarian reserve was considered normal; the preoperative anti-Mullerian hormone level was 5.3 ng/mL. The hysterosalpingography and the partner's spermogram were normal. The MRI showed a hypointense nodular lesion on T1- and T2-weighted sequences measuring 1.6 × 1.3 × 1.0 cm in the left paracolpium. The lesion was described as a deep infiltrative endometriosis nodule (Figures 1 and 2). Laparoscopic resection of the lesion was indicated due to infertility and severe pelvic pain (dysmenorrhea and dyspareunia).

### 2.2. Surgical Technique

No preoperative bowel preparation was performed. Cefoxitin 2 g was administered intravenously immediately prior to anesthesia. The patient was placed in the Lloyd-Davies position on a heated mattress. Elastic stockings and intermittent pneumatic compression of the lower limbs were used for DVT prophylaxis.

A siliconized 8 Fr Foley catheter was introduced into the cervix so that a tubal patency test can be performed later in the procedure. The uterine manipulator was then introduced. After introduction of the first port, the pneumoperitoneum was installed with the CO_2_ pressure set at 10 mmHg. A 10 mm optical with a 30° angle was used. Three 5 mm accessory ports were placed in the left iliac fossa, right iliac fossa, and left flank.

**Figure 3:**
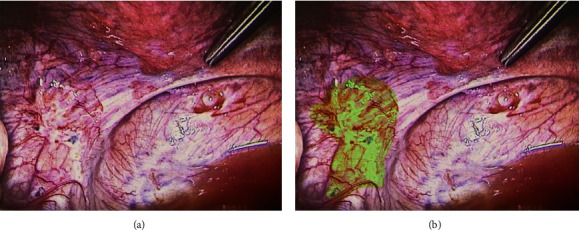
(a) Uterosacral ligament. (b) Same image with “powder-burn” lesions in the left ovarian fossa highlighted in light green.

Cavity inventory was performed in all abdominal quadrants before and after placing the patient in Trendelenburg. In the pelvis, uterosacral ligament thickening and several “powder-burn” lesions compatible with foci of peritoneal endometriosis in the ovarian fossae were observed (Figure 3).

Using Harmonic® ultrasonic shears (Ethicon Endo Surgery Inc, Johnson & Johnson Medical SPA, Somerville, NJ), the sigmoid was mobilized from the level of Toldt's fascia until the identification of the left ureter where it crosses the left common iliac artery. Left ureterolysis was performed from where it crosses the common iliac artery to its intersection with the uterine artery.

The next step was the dissection of the left pararectal space, in order to separate the uterosacral ligament affected by endometriosis from the hypogastric nerve and from the ureter. Dissection of the Latzko space was then performed, with preservation of the left mesoureter. This space is important when approaching the parametrium and paracolpos with endometriotic lesions, as it allows distal control of vascular lesions in these regions and reduces inadvertent lesions to the ureter, pelvic splanchnic nerves, and inferior hypogastric plexus [5].

**Figure 4:**
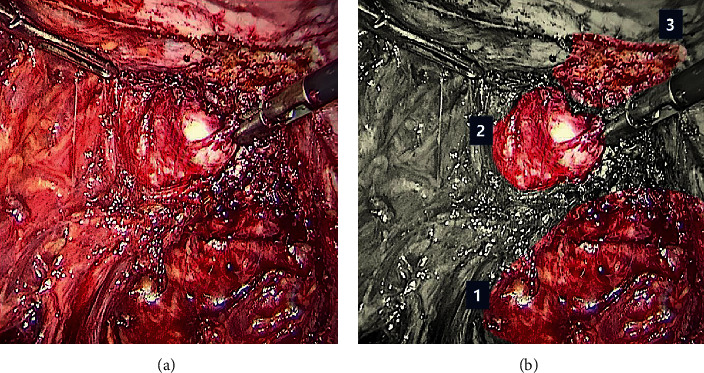
(a) Left paracolpium. (b) Same image identifying (1) left pararectal space, (2) tumor in the left paracolpium, and (3) insertion of the left uterosacral ligament.

During dissection of the left paracolpos, a well-defined hardened region was identified below the cardinal ligament. When the cardinal ligament was incised, a plane of cleavage was observed that usually does not occur with lesions of deep infiltrating endometriosis (Figure 4). This cleavage plane was dissected, and the nodular lesion was removed in its entirety without opening the vaginal mucosa.

Multimodal approaches to management of pain and postoperative nausea and vomiting were implemented. Postoperative analgesia included infiltration of port sites with bupivacaine with epinephrine 0.5% (approximately 20 mL) at the end of surgery and scheduled systemic analgesia with intravenous ketorolac tromethamine and dipyrone.

### 2.3. Postoperative Assessment

The patient tolerated an oral diet 2 hours after surgery. She began walking 4 hours after surgery and thus did not require pharmacological thromboprophylaxis. After spontaneous diuresis, the patient was discharged within 24 hours, with no movement limitation or complaints of pain.

She was followed up with postoperative pelvic physiotherapy as a routine part of the multidisciplinary treatment of deep dyspareunia in our service. At her postoperative review 30 days after surgery, the patient reported that the dysmenorrhea was considerably less intense (NRS = 1 versus 7 preoperatively) and that she had already had sexual intercourse without dyspareunia (NRS = 0).

**Figure 5:**
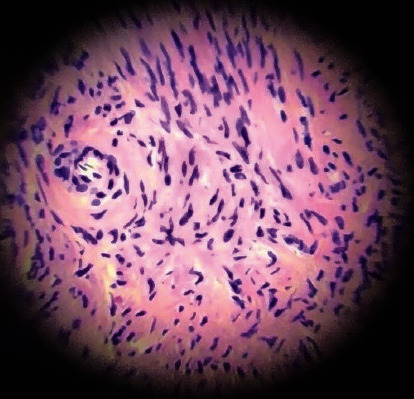
Microscopy of the paracolpal tumor: leiomyoma.

Histopathological evaluation of the paracolpal tumor revealed that it was a leiomyoma (Figure 5). The patient was encouraged to attempt natural conception for 6 months before contemplating fertility treatment. She became pregnant by natural conception 8 months after surgery but experienced a miscarriage in the first trimester.

## 3. Discussion

Vaginal leiomyomas are uncommon, and their symptoms can mimic endometriosis [6, 7]. When indicated, resection of the lesion is usually performed vaginally. In this report, taking into account the initial suspicion of endometriosis, the location, size, and the need to assess another focus of endometriosis in the abdominal cavity, the laparoscopic approach was chosen.

Although both TVUS and MRI are cited as the imaging studies of choice in the diagnosis of leiomyomas and endometriosis [6], MRI has the advantage of evaluating the lateral compartment of the pelvis with greater accuracy [6]. “Leiomyomas are typically round, well-circumscribed, whorl-appearing masses of intermediate T1-weighted and T2-weighted signal intensity, which homogeneously enhance after gadolinium administration [6].”

Our technique for approaching the paracolpal nodule was the same used for the treatment of complex deep endometriosis. This approach makes it possible to prevent injuries to the inferior hypogastric plexus and thus avoid functional complications such as changes in bowel habits, urinary retention, and reduction in vaginal lubrication [5].

As for the postoperative recovery, laparoscopy seems to be related to less postoperative pain compared to vaginal surgery [8]. Measures such as using low pressure in the pneumoperitoneum, encouraging early ambulation, and a careful protocol of postoperative analgesia including infiltration of port sites with a long-acting local anesthetic all seem to contribute to a more comfortable recovery [9, 10].

Egbe et al. described a case of vaginal myomectomy in which the couple interrupted their sexual activity and psychological support was required [4]. Colpotomy can be an important factor in perpetuating or even triggering dyspareunia [11]. In a case series involving patients undergoing surgery to treat endometriosis, Crispi et al. noted that in two-thirds of the cases with unfavorable dyspareunia outcomes, some type of colporrhaphy had been performed. We believe that the laparoscopic approach brought less harm to the patient's sexual life, as it avoided colpotomy and allowed the treatment of coexisting intraperitoneal lesions. The surgical approach employed in this case provided a better visualization of key structures in the pelvis and helped avoid lesions to these structures during resection of the leiomyoma [10, 12].

## 4. Conclusion

When surgical treatment of a paracolpal tumor is indicated, the location of the lesion should be considered, and the laparoscopic approach could be a good option for lesions of the upper third of the vagina. The systematization for the treatment of paracolpal leiomyomas can be the same as that used for nerve sparing deep infiltrative endometriosis resection. The laparoscopic approach to vaginal lesions can minimize functional complications, especially dyspareunia, and favors a rapid recovery. Until the impact of colpotomy on women's sexual is better elucidated, laparoscopic surgery can be an option in the treatment of vaginal leiomyomas which avoids colpotomy.

## Data Availability

All data used to support the findings of this study are included within the article.
